# Overall Survival in Heart Disease–Related Death in Non-Small Cell Lung Cancer Patients: Nonimmunotherapy Versus Immunotherapy Era: Population-Based Study

**DOI:** 10.3389/fonc.2020.572380

**Published:** 2020-10-22

**Authors:** Mohammed Safi, Ravindran Kanesvaran, Mohammed Alradhi, Abdullah Al-Danakh, Feng Ping, Najeeb Al-Sabai, Xiu Shan, Jiwei Liu

**Affiliations:** ^1^Department of Oncology, First Affiliated Hospital of Dalian Medical University, Dalian, China; ^2^National Cancer Center Singapore, Singapore General Hospital, Singapore, Singapore; ^3^Department of Urology, Second Affiliated Hospital of Dalian Medical University, Dalian, China; ^4^Dalian Medical University, Medical School, Dalian, China

**Keywords:** non-small cell lung cancer, immunotherapy, SEER database, cardiotoxicity, heart diseases

## Abstract

The cardiotoxicity during immunotherapy administration leads to mortality by more than 42% and heart disease–related mortality among immunotherapy-linked cancers is still considered to be underestimated. In this study, the advanced stage of non-small cell lung cancer (NSCLC) with heart disease–related death was selected in accordance with immunotherapy approval time. NSCLC was searched on the Surveillance, Epidemiology, and End Results (SEER) program. Results show that 538 advanced NSCLC cases, those dominated by men and elderly people aged more than 70 years, had a high percentage of heart disease–related death in both eras. The difference between contemporary groups was fairly nonsignificant (*P* = > 0.05). The overall survival (OS) of all-cause mortality difference showed improved survival in the immunotherapy group (*P* = 0.0001). In the study of heart disease–related death survival with adjusted data, the NSCLC patients show significant lower survival in the immunotherapy era compared with the nonimmunotherapy era (*P* = 0.003; hazard ratio [HR] = 1.31; 95% CI = 1.099–1.57). In the multivariate analysis of NSCLC-related immunotherapy, histology revealed that the non-squamous cell type had an independent risk for lower OS than the squamous cell type (*P* = 0.04; HR= 0.74; CI = 0, 55- 0.99). The results demonstrate the survival benefits for NSCLC in immunotherapy; however, in heart disease–related death, immunotherapy in patients with NSCLC shows decreased OS. This study highlights that NSCLC patients should be highly monitored during immunotherapy administration, and further assessment is needed.

## Introduction

The U.S. Food and Drug Administration (FDA) has approved several immunotherapeutic drugs for cancer since 2010, and many more are still being evaluated in other clinical studies to remarkably increase the response and survival rates of patients with advanced cancer (1). Unfortunately, cancer immunotherapies possess potential toxicity that is distinctive from other types of care, mostly due to their etiology. The occurrence of cardiovascular adverse events is particularly challenging to cancer management and has led to various clinical outcomes, ranging from cardiogenic shock to death (2).

Improving clinical effectiveness should be weighed against potentially dangerous adverse events when selecting immunotherapy plans and comparatively evaluating each related adverse event separately. Even though the incidence of cardiotoxicity-linked immunotherapy is rare, recent studies have implied its underestimation and needs to be reconsidered. In addition, an urgent intervention must be planned to reduce the mortality rate associated with adverse effects (3, 4). More studies that aim to create thorough risk and etiological stratification models and identify pathways that specify this toxicity clause are needed to improve the early prevention, detection, and treatment approach (5).

Recent studies suggest a particular increased incidence of cardiovascular toxicity in lung cancer patients among all based immunotherapies cancers (6), and the evidence of OS of heart disease–related death for NSCLC patients is still lacking. In addition to the nonimmunotherapy era, this study explains the survival variability of the differences in cardiac-related death among NSCLC patients by using the SEER database.

## Patient and Methods

### Study Cohorts

**Figure 1:**
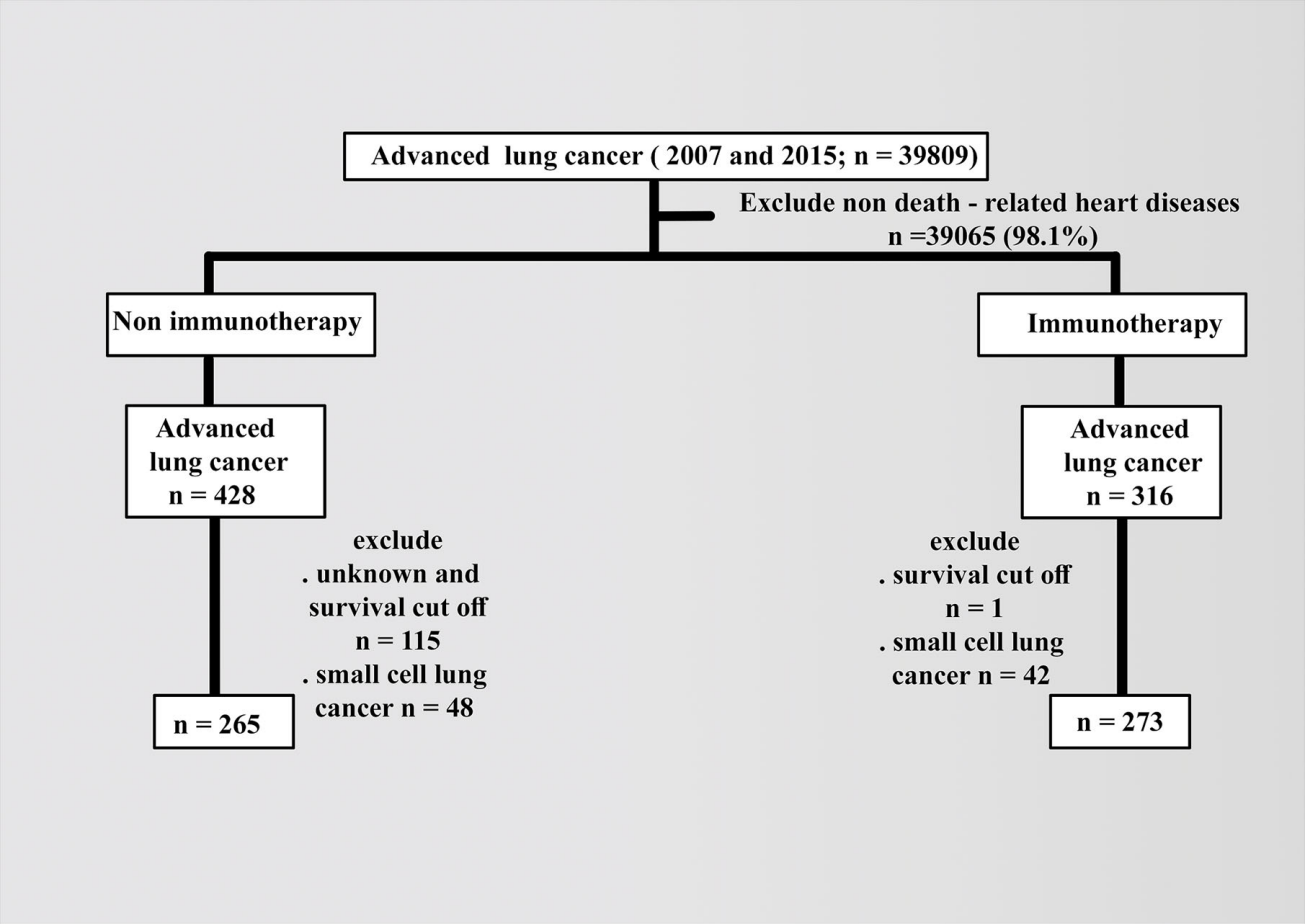
Diagram of exclusion and inclusion criteria.

Patient data were collected from the latest 2018 registry with additional treatment fields on SEER Stat software (version 8.3.6). Using the sixth edition of AJCC, the appropriate codes for advanced lung cancer (IIIB, IV) were selected as labeled site codes (C34/1, 2, 3, and C61.6, respectively). In addition to the period’s equality, the same era of the major targeted therapy of tyrosine kinase inhibitors, 2007, was selected as a year of insurance availability in the database when studying the effects of variables in patients with advanced NSCLC compared with 2015 (7). All patients were designated based on the type of follow-up (active follow-up), and only microscopically confirmed cases (positive histology and positive exfoliative cytology, positive histology and immunophenotyping and/or postgenetic studies, and positive microscopic nonspecified method) were included. The following variables were selected: age (20 years or more), COD to site rec KM, year of diagnosis according to contemporary intervals, ICD-0-3 hist/behav, all survival months, grade (I–II, III–IV, or others), sex (male or female), race (white, black, or others), radiation (radiation or others), chemotherapy (yes or no), vital status record, laterality (right, left, or others), ID patients, marital and insurance statuses (yes or others). The detailed inclusion and exclusion criteria are summarized in **Figure 1**.

In addition to excluding small cell types in advanced lung cancer, patients with heart diseases as the cause of death were included with known survival of 20 months as the cutoff value. The baseline demographics of patients were compared using the x^2^ test, and the follow-up cutoff value was limited to 20 months, depending on the time of advanced NSCLC. Median OS was analyzed using the Kaplan–Meier (KM) method *via* the log-rank test and Cox proportional hazard model for multivariate analysis. Statistical significance was considered at a *p* value less than 0.05 and limits of 0.0001.

## Results

The database contained 538 advanced NSCLC (265 in nonimmunotherapy era vs. 273 in immunotherapy era). The age of over 70 years (e.g., 52% between 75 and 79 years in lung cancer) and male predominance were high in all cases of heart disease–related death in advanced NSCLC, and the distribution of variables was reasonably uniform in both eras (*p* > 0.05). The details of all characteristics are explained in **Table 1**.

**Table 1:** Characteristics of heart disease–related death NSCLC patients.

Parameters	Nonimmunotherapyn = 265(%)	Immunotherapyn = 273(%)	P value
Age			0.6
20-69	98 (37)	107(39.2)	
>70	167(63)	166(60.8)	
Sex			0.7
Male	170(64.5)	179(65.6)	
Female	95(35.8)	94(34.4)	
Race			0.3
White	213(80.4)	205(75.1)	
Black	37(14)	47(17.2)	
Others	15(5.7)	21(7.7)	
Marital status			0.3
Yes	130(49.1)	122(44.7)	
Others	135(50.9)	151(55.3)	
Grade			0.3
I-II	59(22.3)	45(16.5)	
III - IV	67(25.3)	94(34.4)	
Unknown	139(52.5)	134(49.1	
Origin			0.5
Left	116(43.8)	113(41.4)	
Right	149(56.2)	160(58.6)	
Others		1	
Histology			0.002
Adenocarcinoma	96(36.2)	134(49.1)	
Squamous cell cancer	79(29.8)	80((29.3)	
others	90(34)	59(21.6)	
Radiation status			0.8
Yes	97(36.6)	98(35.9)	
No	168(63.4)	175(64.1)	
Chemotherapy			0.8
Yes	105(39.6)	106(38.8)	
No	160(60.4)	167(61.2)	
Insurance			0.8
yes	161(60.8)	168(61.5)	
others	104(39.2)	105(38.5)	

**Figure 2:**
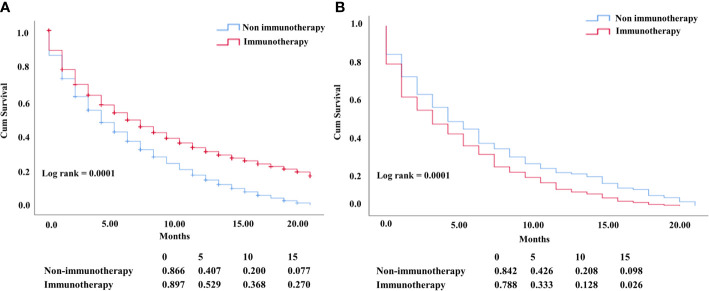
**(A)** KM NSCLC curve comparing OS of all-cause mortality (*P*= 0.0001). **(B)** OS difference in patients who died from heart diseases with negative OS in the NSCLC immunotherapy era (*P* = 0.0001).

The OS difference between the nonimmunotherapy era and the immunotherapy era for advanced NSCLC was statistically significant with the improvement in median survival of the immunotherapy groups (*P* = 0.0001; median survival: 4 vs. 6 months) (**Figure 2A**). By contrast, when considering only heart disease–related death, the OS in the nonimmunotherapy era was significantly better in advanced NSCLC than that in the immunotherapy era (5 months OS 42% vs. 0.33%; 10 months OS months 21% vs. 13% and median survival 4 vs. 3 months; *P* = 0.0001), indicating the negative effect of immunotherapy (**Figure 2B**).

**Figure 3:**
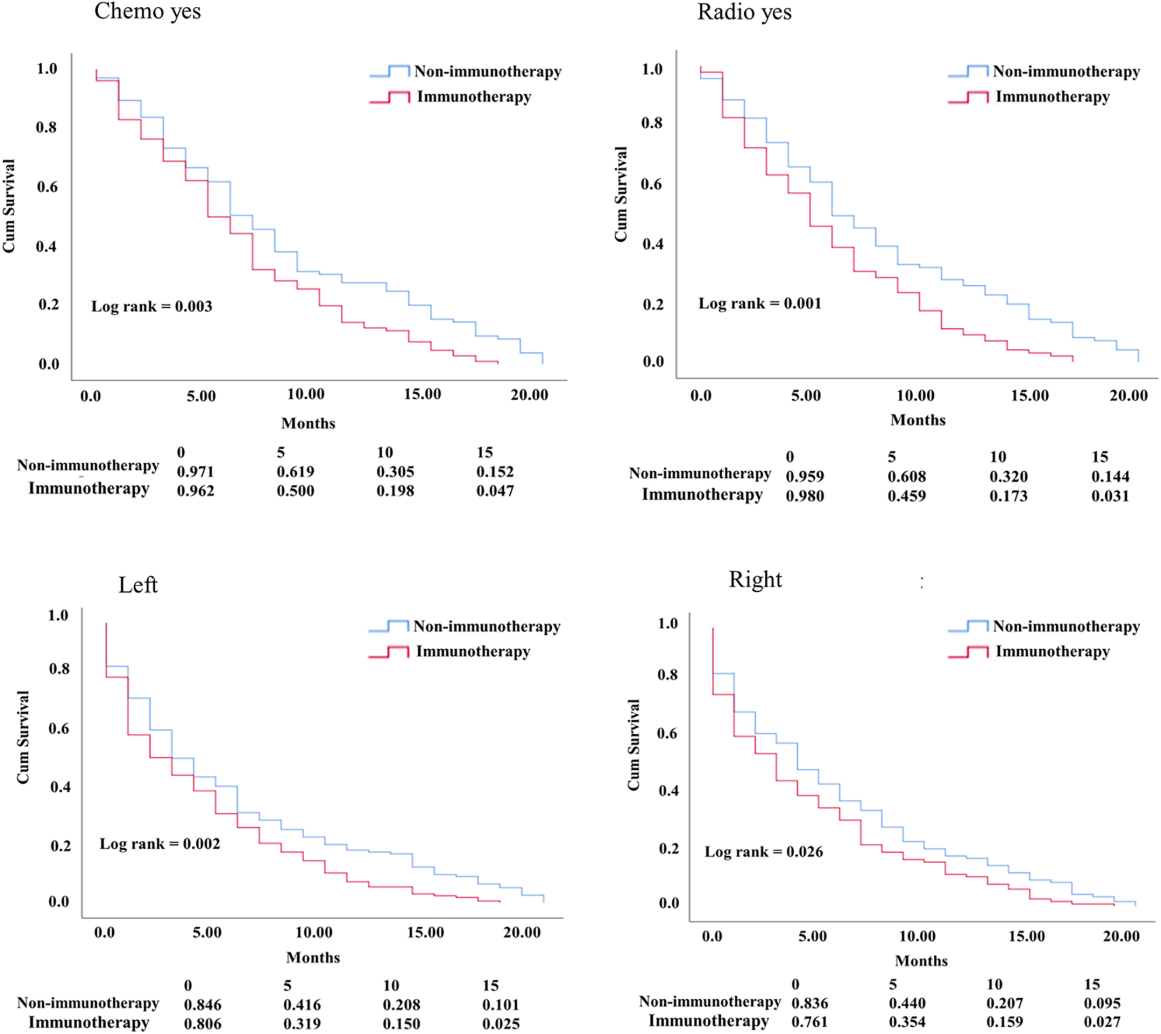
KM curve comparing OS. Factors difference between NSCLC nonimmunotherapy versus immunotherapy era.

**Figure 4:**
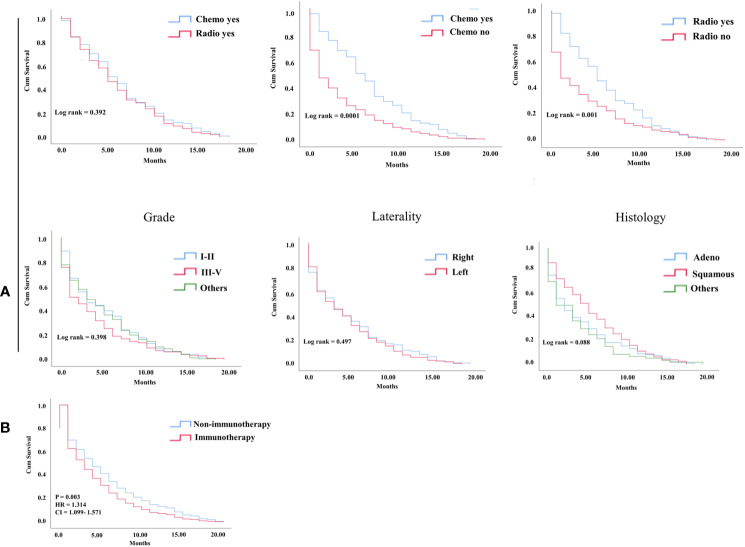
**(A)** KM curve showing OS difference among factors in the NSCLC immunotherapy group of hear related- death patients. **(B)** Cox multivariate analysis difference survival of heart related death patients between NSCLC nonimmunotherapy group and immunotherapy group.

In the KM study of NSCLC difference in OS between the variables of nonimmunotherapy and immunotherapy eras, older age and III–IV grades showed a significant difference in median OS (4 vs. 3 months, 4 vs. 2 months, respectively). In addition, a difference was observed among most other variables with survival benefits restricted to patients in the nonimmunotherapy era (e.g., male, female, white, black, laterality, chemotherapy, and radiotherapy status) (**Figure 3**, **Table 2**). The OS in the immunotherapy era was studied, and the results reveal that chemotherapy and radiotherapy use had beneficial significance to OS (*P*= 0.0001, *P* = 0.001, respectively) with no preferences of each on other (*P* = 0.392). In addition, evidence of OS difference was not found in grade, histology, or laterality (*P* = 0.39, 0.08, 0.49, respectively) (**Figure 4A**). The negative OS of heart disease–related death in the immunotherapy era was still significantly evident after adjusting the data for age, sex, marital status, race, chemotherapy and radiation status, grade, laterality, histology, and insurance status by using Cox regression multivariate analysis (*P* = 0.003; hazard ratio [HR] = 1.31; 95% CI = 1.099–1.57) (**Figure 4B**).

**Table 2:** Survival pattern in heart disease–related death NSCLC patients.

Parameters	Nonimmunotherapy	Immunotherapy	*P* value bylog rank test
Age	median OS (months)	median OS (months)	
20-69	4	4	0.08
>70	4	3	0.001
Sex			
Male	4	3	0.007
Female	5	3	0.007
Race			
White	4	3	0.004
Black	5	3	0.03
Others	4	3	0.1
Marital status			
Yes	4	3	0.002
Others	4	3	0.02
Grade			
I-II	5	3	0.06
III-IV	4	2	0.006
Others	4	3	0.07
Unknown			
Left	4	3	0.02
Right	4	3	0.002
Missed 1			
Histology/behave			
Adenocarcinoma	3	2	0.2
Squamous cell cancer	4	5	0.1
Others	6	3	0.0002
Radiation status			
Yes	6	5	0.001
No	2	1	0.01
Chemotherapy			
Yes	7	5	0.003
No	2	1	0.006
Insurance status			
Yes	5	3	0.003
No/unknown/others	4	2	0.01

The multivariate analysis of advanced lung cancer in the immunotherapy era revealed that the nonsquamous type was significant and showed worse survival of cardiac disease–related death patients than the squamous type (HR = 0.74; 95% CI = 0.55–0.99; *P* = 0.04). Chemotherapy use was not associated with poor OS in cardiac disease–related death (*P* = < 0.0001; HR= 1.812; CI = 1.37-2.38). Radiotherapy use in advanced lung cancer improved the OS in the nonimmunotherapy era but did not predict the OS in the immunotherapy era, mostly because of the large effect of chemotherapy use in this group, as shown in a separate model (*P* = 0.005; HR = 1.47; 95% CI = 1.12–1.93 vs. *P* = 0.06; HR = 1.29; 95% CI = 0.98–1.70) (**Table 3**).

**Table 3:** Univariate and multivariate analysis of factors affecting the survival in heart disease–related death NSCLC patients.

Parameters	Nonimmunotherapy				Immunotherapy			
	Univariate HR/CI	P value	Multivariate HR/CI	P value	Univariate HR/CI	P value	Multivariate HR(95% CI)	P value
Age (20-69 vs. >70)	1.06(0.82-1.36)	0.6	0.76(0.58-1.00)	0.05	0.92(0.72-1.17)	0.5	1.00(0.77-1.29	0.9
Sex Male vs. Female	1.07(0.83-1.38)	0.5	0.87(0.57-1.22)	0.3	1.03(0.80-1.33)	0.7	0.88 (0.67-1.16	0.3
Marital Yes - Others	0.84(0.66-1.07)	0.1	1.06(0.81-1.38)	0.6	0.93(0.73-1.18)	0.5	0.96(0.75-1.26)	0.8
Race								
White	Reference		Reference		Reference		Reference	
Black	0.81(0.57-1.15)	0.2	0.83(0.57-1.22)	0.3	0.97(0.70-1.34)	0.8	0.85(0.60-1.19)	0.3
Others	0.56(0.32-0.98)	0.04	0.51(0.28-0.91)	0.02	0.99(0.63-1.55)	0.9	0.82(0.51-1.30)	0.4
Chemotherapy yes vs. no	0.57(45-74)	<0.0001	1.72(1.31-2.25)	0.0001	1.76(1.38-2.26)	0.0001	1.81(1.37-2.38)	0.0001
Radiotherapy yes vs. no	0.61(48-0.79)	0.0001	1.47(1.12-1.93	0.005	1.46(1.13-1.87	0.003	1.29(0.98-1.70)	0.06
Grade								
I-II	Reference		Reference				Reference	
III-IV	1.14(0.80-1.62)	0.4	1.28(0.89-1.84	0.1	1.20(0.84-1.71)	0.3	1.24(0.86-1.79)	0.2
Unknown	1.30(0.95-1.77		1.23(0.88-1.72)	0.2	1.04(0.74-1.46)	0.8	1.05(0.73-1.49)	0.7
Laterality Left vs. Right	0.99(0.77-1.26)	0.9	1.02(0.79-1.32)	0.8	0.93(0.73-1.18)	0.5	1.21(0.93-1.57)	0.1
Histology/Behav								
Adenocarcinoma	Reference						Reference	
Squamous	0.77(0.57-1.04)	0.094	0.78(0.56-108)	0.1	0.80 (0.61-1.06)	0.133	0.74(0.55-0.99)	0.04
Others	0.71(0.053-0.09)	0.022	0.70(0.52-0.95)	0.02	1.12 (0.82-1.52)	0.474	0.96 (0.69-1.35)	0.8
Insurance yes vs. others	0.85(0.66-1.10)	0.228	1.07(0.81-1.41	0,6	1.21 (0.95-1.55)	0.114	1.25(0.96-1.61)	0.08

## Discussion

The presence of baseline organ dysfunction in patients on immune check inhibitors demonstrates general immune adverse events similar to those in previous clinical trials that included patients without organ dysfunction (8). Although it is representative of less than 1% of adverse immune events, immune inhibitor-caused myocarditis is a potentially fatal condition associated with 42% mortality (9). However, in general immunotherapy-related cardiotoxicity symptoms, several studies suggest an increase in patients with end organ failure compared with those without organ dysfunction (in both cases of myocarditis or controls without myocarditis) (10). The median time to the presentation of cardiotoxicity-related immunotherapy ranged from 2 to 454 days (23 months), and the majority occurred within the first four cycles of immunotherapy (1st month) (11). To the best knowledge of the authors, the present study was a large unique study that detected the OS of patients who died from heart diseases in the contemporary eras of immunotherapy and nonimmunotherapy NSCLC.

In this decade, many reported clinical trials for immunotherapy in various solid cancer types, including earlier challenging cancers, revealed an increase in OS and considerable strength in the treatment of NSCLC, in either combination or monotherapy (12, 13). The present study, which is a population-based study that used the SEER database, reveals good survival benefits in advanced NSCLC, which were largely attributable to the introduction of immunotherapeutic drugs to therapeutic regimens. Where the median OS increased from 4 to 6 months, this improvement was distinctly lower than those reported by related approved studies (14), However, the latter was predominantly related to the cutoff value for survival with only 20 months in all age groups regardless of their performance status and other comorbidities.

In the study of OS in patients with heart disease–related death, the OS was significantly decreased in the NSCLC immunotherapy era compared with the nonimmunotherapy era. The remaining patients in the first 5 months of survival from NSCLC were markedly lower in the treatment-based immunity era than in the nonimmunity-based era (33% vs. 42%). This finding could be explained by the following: 1) An association between high cardiotoxicity-related mortality rate and immunotherapy exists (15). 2) Regarding the relationship of cardiotoxicity and immunotherapy, most patients present their symptoms shortly after treatment (16). 3) The risk of incidence of major cardiac adverse events is increased in thoracic tumors (17). Except for adenocarcinomas and squamous cell histology of lung cancer, most variables demonstrate significant negative OS in the NSCLC immunotherapy era. In addition, aging is significantly linked to negative OS in patients with NSCLC who died from heart diseases; this finding was also recently shown in a SEER-Medicare study by Bora et al., who report that comorbidities and negative OS are related to old age in NSCLC patients who were started with immune checkpoint inhibitors (18). Recently, several reports indicate that the combining of chemoradiotherapy with immunotherapy has superior efficacy in producing improved anticancer activity (19). In the immunotherapy group of heart disease–related death, chemotherapy and radiotherapy use as monotherapy in advanced NSCLC exhibit improved OS compared with nonuse. In the study difference between radiotherapy and chemotherapy use, no survival significance has been seen. Thereby, the risk of decreased OS in this group was more independently associated with the use of immunotherapy than the synergetic negative effect of other interventions.

Cox multivariate analysis confirms that immunotherapy is a risk predictor for OS in patients who died from heart diseases in the NSCLC immunotherapy era (HR= 1.314; *P* = 0.003). Even though it was significant in older age based on KM, the difference in age groups demonstrated by the Cox model in the new era of NSCLC did not provide any benefits of survival. In a multicenter retrospective study of the association between age with immune-related events and OS in NSCLC, age was not an independent risk factor of survival (20). Chemotherapy and radiotherapy use was significantly observed in the nonimmunotherapy era as a high predictor of increased OS. By contrast, the use of chemotherapy significantly decreased the risk of heart disease–related death. Radiotherapy use was not a risk factor for survival differences in the immunotherapy era, mostly due to the substantial effect of newly developed targeted therapies in recent years. The histology of squamous cancers demonstrated a prominent significance with positive survival benefits compared with nonsquamous cancers, indicating that histology could be a protective factor. Another explanation considers it could be the mortality from cancer and its poor prognosis compared with other types of histology (21, 22).

This study has several limitations. First, the SEER database did not provide an explanation about heart disease as the cause of death in patients as either real incidents before or newly related immunotherapeutic events. Second was the short median OS associated with the 20-month follow-up, the comorbidities, and the lack of performance status information. Third, the study lacked a detailed description of immunotherapy for patients.

## Conclusion

This study demonstrates the OS benefits for NSCLC patients in the immunotherapy era compared with that in the nonimmunotherapy era that was primarily attributed to the immunotherapy. In heart disease–related death, immunotherapy in patients with NSCLC demonstrated decreased OS. Chemotherapy use increased the OS in patients with lung cancer who died from cardiac diseases, whereas no OS difference was found in radiotherapy use. Also, recognition of histology during immunotherapy, especially for nonsquamous types, could be considered as another predictor of OS reduction in patients who died from heart disease during immunotherapy. Although the incidence of cardiac toxicity is less than 1%, the risks must be assessed in all elderly patients with NSCLC. This study strongly highlighted effective clinical and preclinical studies to enhance the results.

## Data Availability Statement

All datasets presented in this study are included in the article/supplementary material.

## Ethics Statement

Written informed consent was obtained from the individual(s) for the publication of any potentially identifiable images or data included in this article.

## Author Contributions

Conceptualization: MS. Validation: MS and XS. Formal Analysis: MS, JL, MA, and AA-D. Investigation, writing, original draft preparation: MS. Visualization: FP, MA, AA-D and NA-S. Supervision: JL, and RK. Project administration: MS and JL. All authors contributed to the article and approved the submitted version.

## Funding

National Natural Science Foundation of China, Grant/Award Numbers: 81602508, 81572881, 31770859.

## Conflict of Interest

The authors declare that the research was conducted in the absence of any commercial or financial relationships that could be construed as a potential conflict of interest.
